# Shear-Wave Elastography for Predicting the Occurrence and Assessing the Severity of Capsular Contracture After Implant-Based Breast Augmentation and Reconstruction: A Review of the Evidence

**DOI:** 10.3390/jcm15218408

**Published:** 2026-10-28

**Authors:** Mihai Iliescu-Glaja, Fabiana Simion, Andrei Motoc, Daciana Grujic, Cristi Tarta, Dana Stoian

**Affiliations:** 1 Doctoral School, “Victor Babes” University of Medicine and Pharmacy, E. Murgu Square, No. 2, 300041 Timisoara, Romania; mihai.iliescu.glaja@umft.ro; 2 Plastic and Reconstructive Surgery Department, Pius Brinzeu Clinical County Emergency Hospital Timisoara, Casa Austria, Liviu Rebreanu Blvd 156, 300723 Timisoara, Romania; grujic.daciana@umft.ro; 3 Department of Anatomy and Embryology, Faculty of Medicine, “Victor Babes” University of Medicine and Pharmacy, E. Murgu Square, No. 2, 300041 Timisoara, Romania; amotoc@umft.ro; 4 Plastic Surgery Department, “Victor Babes” University of Medicine and Pharmacy, E. Murgu Square, No. 2, 300041 Timisoara, Romania; 5 Center for Advanced Research in Cardiovascular Pathology and Hemostaseology, “Victor Babes” University of Medicine and Pharmacy, E. Murgu Square, No. 2, 300041 Timisoara, Romania; 6 Researching Future Surgery II Research Center, Department X, Discipline of General Surgery II, Faculty of Medicine, “Victor Babes” University of Medicine and Pharmacy, E. Murgu Square, No. 2, 300041 Timisoara, Romania; 7 Department of Internal Medicine II, Discipline of Endocrinology, “Victor Babes” University of Medicine and Pharmacy, E. Murgu Square, No. 2, 300041 Timisoara, Romania; stoian.dana@umft.ro

**Keywords:** shear-wave elastography, ultrasound elastography, capsular contracture, breast implant, breast augmentation, breast reconstruction, Baker classification, periprosthetic capsule, tissue stiffness

## Abstract

Capsular contracture (CC) is the most common long-term complication of implant-based breast augmentation and reconstruction, yet it is still graded by palpation with the Baker classification, whose interobserver reliability is poor. Shear-wave elastography (SWE) quantifies tissue stiffness non-invasively and could assess the periprosthetic capsule objectively. We searched bibliographic and publisher databases up to September 2026 and critically appraised every primary clinical report applying ultrasound elastography to the periprosthetic capsule. Eight reports (2011–2026; 2–51 patients each) from four groups were identified; seven used shear-wave techniques and one compression elastography. Wherever high-grade contracture was represented, periprosthetic stiffness increased with the Baker grade. After reconstruction, SWE was more reproducible than strain elastography and correlated more closely with the Baker score (r = 0.81 versus 0.70). In one comparative study, stiffness almost completely separated Baker III/IV reconstruction breasts from Baker I/II augmentation breasts (area under the curve 0.994), but the groups also differed in indication, age and protocol, so diagnostic accuracy cannot be inferred; neither proposed threshold (~52 and 82 kPa) has been externally validated. Longitudinal studies show early postoperative stiffening that partly normalizes within a year, but none has shown that early SWE predicts subsequent contracture. SWE is a promising adjunct to clinical assessment, not a stand-alone criterion for surgery; standardized acquisition, indication-matched validation and prospective predictive studies are required before clinical adoption.

## 1. Introduction

Implant-based breast surgery, for cosmetic augmentation and for reconstruction after mastectomy, is among the most frequently performed procedures in plastic surgery, and capsular contracture (CC) remains its most common long-term local complication [1,2]. Every implanted device becomes enveloped by a fibrous capsule; CC denotes the pathological thickening and contraction of this capsule, which produces firmness, distortion and displacement of the implant and, in advanced cases, pain [1]. Long-term data from the United States core clinical studies illustrate the size of the problem. In the Natrelle round silicone implant Core Study, the 10-year Kaplan–Meier rates of CC were 18.9% after primary augmentation, 28.7% after revision augmentation and 24.6% in the reconstruction cohort [3]; in the MemoryGel Core Study, the 10-year rates of Baker grade III/IV contracture were 12.1% for primary augmentation, 24.4% for revision augmentation, 20.5% for primary reconstruction and 36.9% for revision reconstruction [4]; and in the Natrelle 410 anatomical form-stable implant Core Study the corresponding 10-year rates were 9.2% (augmentation), 11.9% (revision augmentation), 14.5% (reconstruction) and 26.8% (revision reconstruction) [5]. In the reconstructive setting, post-mastectomy radiotherapy (PMRT) is the dominant modifier of risk: a meta-analysis of immediate prosthetic reconstruction found that PMRT increased the odds of CC more than five-fold (odds ratio 5.26; 95% confidence interval [CI] 2.73–10.13) [6], and in a contemporary series of 118 women who received PMRT after implant-based reconstruction, 22.9% developed Baker grade III–IV contracture and 47.5% developed contracture of any grade [7].

Despite its clinical importance, CC is still diagnosed and graded almost exclusively by palpation, using the four-grade scale introduced by Baker in the 1970s [8] and later adapted by Spear and Baker for the reconstructed breast [9] (Table 1). The subjectivity of this scale has been quantified. In a study in which two plastic surgeons independently graded 60 women with breast implants, the interobserver reliability of the Baker classification was poor (quadratic weighted kappa 0.55; 95% CI 0.37–0.72), with exact agreement in only 48% of cases, and the authors concluded that the classification is unreliable as a diagnostic tool [10]. A systematic review published in 2024 identified 19 articles proposing objective approaches to the assessment of CC—magnetic resonance imaging (MRI), ultrasonography, sonoelastography, mammary compliance devices, applanation tonometry, histology and serology—and concluded that no single method reliably and specifically measures the contracture of the capsules that form around breast implants, recommending that more than one modality be used [2].

Shear-wave elastography (SWE) is a quantitative ultrasound technique that estimates tissue stiffness from the propagation speed of shear waves generated by an acoustic radiation force impulse [11,12]. Because fibrosis and collagen deposition increase tissue stiffness, and because CC is fundamentally a fibrotic process of the periprosthetic capsule, SWE is an intuitively attractive candidate for an objective, non-invasive and widely available measure of capsule mechanics. SWE is well established for the characterization of breast masses and is the subject of international guidelines [13,14], but its application to the periprosthetic capsule has been explored in only a small number of studies.

The aim of this review is to synthesize, critically and without overstatement, the available evidence on the use of SWE: (i) to assess the severity of established CC and (ii) to predict the future occurrence of CC after implant-based breast augmentation and reconstruction. We also summarize the physical principles and technical constraints specific to imaging a thin fibrous layer adjacent to a silicone implant, compare SWE with other objective methods, identify the limitations of the current evidence base and propose priorities for future research.

## 2. Materials and Methods

### 2.1. Search Strategy

This is a narrative review with a structured literature search. It was not registered as a systematic review, and no meta-analysis was attempted because of the small number and the methodological heterogeneity of the primary studies (Section 2.3). We searched PubMed/MEDLINE, PubMed Central, Europe PMC, Google Scholar and the databases of the relevant publishers (IOS Press/SAGE, Springer Nature, Elsevier/ScienceDirect, Wolters Kluwer, Oxford Academic, MDPI and Termedia) from database inception to September 2026, without language restriction, using combinations of the terms “shear wave elastography”, “shear-wave”, “sonoelastography”, “ultrasound elastography”, “acoustic radiation force impulse”, “ARFI”, “Young’s modulus”, “stiffness” and “elasticity” with “capsular contracture”, “capsular fibrosis”, “periprosthetic capsule”, “breast implant”, “mammary implant”, “silicone implant”, “breast augmentation”, “augmentation mammaplasty”, “breast reconstruction”, “implant-based reconstruction” and “Baker classification”. The reference lists of all retrieved articles and of relevant reviews were screened, and forward citation tracking (the “cited by” functions of Google Scholar, PubMed Central and Semantic Scholar) was performed for every included study. ClinicalTrials.gov was searched for registered trials that use elastography as an outcome measure for CC.

### 2.2. Eligibility Criteria and Data Extraction

We included primary clinical reports, of any design and sample size, in which an ultrasound elastography technique (two-dimensional SWE, point SWE/ARFI quantification, or compression/strain elastography) was applied to the periprosthetic capsule or peri-implant tissues of patients with breast implants, after either aesthetic augmentation or reconstruction, and in which the elastographic findings were related to a clinical endpoint (Baker grade, pain or the perioperative course). Studies that applied elastography exclusively to the implant gel (for example, for the assessment of rupture) or to native breast lesions, and studies that used MRI or other non-ultrasound modalities, were excluded from the primary evidence set but were considered as background or comparator literature. From each included study we extracted the country, design, number of patients and breasts/implants, indication, implant characteristics, time since implantation, elastography technique and device, measurement site and parameter, reference standard and principal quantitative results.

### 2.3. Results of the Search

Eight primary clinical reports published between 2011 and 2026 met the inclusion criteria. Seven used shear-wave-based techniques (two-dimensional SWE, with or without additional ARFI or strain measurements) and one used compression (strain) elastography only. The individual reports included between 2 and 51 patients, and their sample sizes sum to 160 analyzed patients. The study populations are not all independent, however: the two earliest series of the Poznań group [15,16] share their setting, surgical period and early examinations, and the 13 patients of the later report were probably among the 19 patients of the earlier one, although neither report states this (Section 5.2). If they were, the literature represents 147 rather than 160 individual patients. To our knowledge, no animal or ex vivo study of capsule elastography has been published; three experimental elastography studies of the implant itself were identified, two of which are discussed in Section 3 [17,18] and one, in an animal model, in Section 9. One registered trial using SWE as an outcome measure for CC was identified [19]. Two further clinical SWE studies in augmentation patients, one measuring native tissue stiffness before surgery to predict lower pole expansion and one measuring the glandular parenchyma of augmented breasts in relation to mastalgia, did not concern the periprosthetic capsule or CC, did not meet the inclusion criteria and are considered in Section 9.

## 3. Principles of Shear-Wave Elastography and Technical Considerations Around Breast Implants

### 3.1. Shear-Wave and Strain Elastography

SWE belongs to the family of dynamic elastography methods. A focused acoustic radiation force impulse (“push”) displaces tissue by a few micrometers and generates transversely polarized shear waves that propagate laterally at a speed (c_s_) of the order of 1–10 m/s, several orders of magnitude slower than the compressional ultrasound wave; ultrafast plane-wave imaging tracks the wavefront and the local shear-wave speed is computed [11,12,20]. Under the assumptions of a linear, isotropic, homogeneous and quasi-incompressible medium, the shear modulus is μ = ρc_s_^2^ and the Young’s modulus is E ≈ 3ρc_s_^2^ (ρ, tissue density ≈ 1000 kg/m^3^), so that stiffness can be expressed either as a speed (m/s) or as an elastic modulus (kPa) [13,21,22]. Two-dimensional SWE produces a color-coded quantitative elastogram over a region of interest (ROI) in real time, whereas point SWE/ARFI quantification returns a single shear-wave speed from a small fixed measurement box [13,21].

Strain (compression) elastography is, by contrast, a quasistatic method: the tissue is deformed by manual compression or by physiological motion, and the resulting relative strain is displayed as a color map or expressed as a strain ratio with respect to a reference tissue. Because the applied stress is unknown, the measurement is qualitative or semiquantitative and operator-dependent [13,21,22]. This distinction matters for the capsule literature: the first German study used color-coded compression elastography [23], whereas the Polish, Japanese and Romanian studies used quantitative SWE [15,16,24,25,26,27], and the Japanese study was in combination with strain elastography [27]; the second German study combined strain elastography, SWE and ARFI quantification [28].

In breast masses, SWE measurements have been shown to be highly reproducible within and between observers [29], and the technique is endorsed for lesion characterization by the guidelines of the World Federation for Ultrasound in Medicine and Biology (WFUMB) and of the European Federation of Societies for Ultrasound in Medicine and Biology (EFSUMB) [14,22]. Two practical caveats emphasized in these guidelines apply directly to implants: stiffness values increase with transducer precompression, which must therefore be minimized and standardized [30], and absolute values in kPa are not fully interchangeable between manufacturers, so that reporting the shear-wave speed alongside the elastic modulus is recommended [13,22].

### 3.2. Specific Constraints of the Periprosthetic Capsule

Imaging the periprosthetic capsule with SWE raises several issues that the general breast elastography literature does not address (Figure 1).

First, the capsule is thin. Ultrasound measurements in patients with implants reported mean capsule thicknesses of 0.58 ± 0.11 mm, 1.07 ± 0.16 mm and 1.89 ± 0.55 mm in Baker grade II, III and IV breasts, respectively (the capsule of Baker grade I breasts was too thin to be measured on ultrasound) [31], whereas the ROIs used in the capsule SWE studies measured 1–3 mm [27], 2 mm [24] and 2–4 mm [25], and the ARFI measurement window was fixed at 10 mm [28]. In most configurations the ROI therefore averages the capsule with adjacent fat, gland or muscle and with the implant surface; the measured value is a periprosthetic composite stiffness rather than a true capsule modulus, and it can be altered by anything that changes the stiffness of those tissues, independently of the capsule (see Section 6 and Section 8). Moreover, the assumption of an unbounded homogeneous medium does not hold in a thin layer bounded by media with very different mechanical properties: in thin plates, shear waves propagate as dispersive guided waves whose apparent speed depends on frequency and on the thickness of the plate, as has been demonstrated for the arterial wall [32]. The extent to which this affects periprosthetic measurements has not been studied.

Second, the implant itself is not soft tissue. Strain elastography of explanted implants has been used experimentally to characterize the gel [17], and ex vivo shear-wave measurements of explanted implants have shown that the stiffness of the gel increases with implantation time [18]. These studies concern the device rather than the capsule, but they indicate that the shear-wave field close to the tissue–implant interface is influenced by the implant. Experimental data on elastography of the implant itself are considered further in Section 9. Two reports from the same group commented on technical feasibility, noting no problems with shear-wave penetration, including at the surface of the prosthesis [15,26]. Three studies excluded breasts or images of inadequate quality—a non-detectable capsule [27], images without a measurable SWE signal [25] and images of insufficient elastographic quality [28]—but none reported the number of technically failed or excluded acquisitions.

Third, depth: shear-wave generation and tracking degrade with depth, and linear transducers typically provide reliable measurements only within the first few centimeters [13,21]. The capsule of a subpectoral implant in a large breast may lie close to the limit of this range, and the anterior capsule is far more accessible than the posterior capsule, which has not been measured in any published study.

Fourth, the reference range of native breast tissue is itself variable. In 101 women without implants, the mean stiffness of glandular and fatty tissue measured with SWE was 11.28 ± 5.79 kPa and 9.24 ± 4.48 kPa, respectively, with wide ranges, and the stiffness of glandular tissue increased with age (Spearman coefficient 0.27) [33]; in smaller series, the same group found no significant change in the stiffness of glandular or fatty tissue during the menstrual cycle [34] and no correlation of stiffness with estrogen, follicle-stimulating hormone, prolactin or thyroid hormone levels, apart from a weak negative association between androgens and the glandular-to-fat stiffness ratio [35]. Periprosthetic values must therefore be interpreted against the interindividual variability of the surrounding tissues.

## 4. Evidence on Shear-Wave Elastography for Assessing the Severity of Capsular Contracture

### 4.1. Overview of the Primary Studies

Table 2 summarizes the design and methods of the eight primary reports, Table 3 lists their principal quantitative results and main limitations, and Figure 2 maps the studies over time by indication, technique and sample size. The evidence originates from four groups: Poznań, Poland (four reports, 2011–2025) [15,16,24,26]; Regensburg, Germany (two reports, 2014 and 2021) [23,28]; Kyoto, Japan (one report, 2017) [27]; and Timișoara, Romania (one report, 2026) [25]. No study was multicenter, and none included a prespecified validation cohort.

### 4.2. Compression (Strain) Elastography: The First Correlation with the Baker Score

Prantl and colleagues examined 17 smooth silicone gel implants in 11 patients aged 37–53 years (seven bilateral aesthetic augmentations and unilateral reconstructions for mammary asymmetry, numbering four according to the abstract and three according to the Methods, only the latter being consistent with 17 implants) with color-coded compression elastography on a Siemens Acuson Antares Premium system using a 5–10 MHz multifrequency transducer [23]. The implants (125–325 cc) were subpectoral and were examined between 1 week and 6 years after implantation, an interval that the authors acknowledged to begin early for the detection of contracture. Two breast radiologists, unaware of the Baker grade, performed the elastography, and two examiners assigned the Baker grade by palpation in consensus; six implants were Baker I, six Baker II, three Baker III and two Baker IV. A four-grade elastography score based on the stiff margin around the implant (absent, discontinuous, continuous and <3 mm, or >4 mm) and a high-resolution B-mode score (0–5) were compared with the Baker grade. The abstract summarizes the result as a high correlation between the Baker score and the elastography score (kappa 0.83–0.89); in the full text, however, Cohen’s kappa of 0.83 describes the agreement between the two radiologists, whose grades differed in two implants, whereas the elastography grade differed from the Baker grade in four of the 17 implants (23.5%), with a Pearson correlation coefficient of 0.89 [23]. The study established the feasibility of the approach but, being based on operator-applied compression, provided no absolute stiffness values.

### 4.3. Shear-Wave Versus Strain Elastography After Breast Reconstruction

The first direct comparison of strain and shear-wave elastography in this setting was performed by Sowa and colleagues in 20 patients (aged 24–68 years) with 27 implants after two-stage expander–implant breast reconstruction; breasts with a non-detectable capsule and patients with previous breast surgery or radiotherapy were excluded [27]. Both techniques were performed on a GE LOGIQ E9 system with a 4–9 MHz linear probe: strain elastography yielded a strain ratio, and SWE the mean elastic modulus in a circular ROI of 1–3 mm placed on the capsule. The results were compared with the Baker score assigned by two plastic surgeons (grade I, 6 breasts; II, 12; III, 5; IV, 4). The reproducibility of three measurements made by the same operator on the same day was higher for SWE than for strain elastography (intraclass correlation coefficient [ICC] 0.878 [95% CI 0.785–0.937] versus 0.724 [95% CI 0.552–0.851]); interobserver variability was not assessed. The correlation between the two techniques was moderate (r = 0.6788; described as low by the authors), and the Baker score correlated more closely with SWE (r = 0.8124) than with strain elastography (r = 0.6983) [27]. From histograms of the SWE values, the authors suggested a cut-off of approximately 52 kPa for distinguishing breasts with contracture (Baker III/IV) from those without, but reported neither its sensitivity nor its specificity. The authors concluded that SWE is the better tool for assessing the degree of capsular contracture after implant-based reconstruction.

### 4.4. Elastography After Exchange to Polyurethane-Coated Implants

Jung and colleagues followed 16 patients (23 implants according to the abstract, 25 according to the Methods) who had undergone implant exchange to polyurethane-coated implants because of prior CC, the initial implantation having been aesthetic in seven and reconstructive in nine, and examined them in the third year after the exchange with strain elastography, SWE and ARFI quantification (Siemens Acuson S3000 and GE LOGIQ E9 systems; four quadrants and the subareolar region), in addition to the Baker score and a visual analogue pain scale [28]. Pain decreased from 4.1 ± 2.8 before the exchange to 1.7 ± 1.0 at follow-up (*p* = 0.002). The abstract reports a close correlation between ARFI values and pain (r = 0.873); in the Results, however, this coefficient describes the correlation between ARFI values and the Baker score (R = 0.873), whereas the correlation between ARFI values and pain was R = 0.648 (*p* = 0.05) and that between SWE values and the Baker score R = 0.754. Mean ARFI velocities were 2.1, 2.3, 2.6 and 2.5 m/s in Baker I, II, III and IV breasts, respectively, and velocities above 2.3 m/s, a threshold adopted from the staging of liver fibrosis, were regarded as indicating periprosthetic fibrosis. The authors nevertheless stated that elastography correlated only to a limited extent with the Baker findings, and the stiffness of glandular tissue did not differ between implanted breasts and seven contralateral breasts without implants [28]. They considered elastography a promising objective means of classifying capsular fibrosis and called for a prospective multicenter study. This report thus describes a positive but imperfect relationship between elastographic stiffness and the Baker grade after revision surgery; the discrepancies between its abstract and its Results should be borne in mind when its coefficients are cited.

### 4.5. Two-Dimensional Shear-Wave Elastography as a Diagnostic Test for Baker III/IV Contracture

The largest and most recent study is a single-center comparative study from Timișoara, Romania, reported according to the STROBE recommendations [25]; the study group was enrolled prospectively, whereas the control examinations had been acquired during routine follow-up and were included retrospectively. Fifty-one patients were enrolled: a control group of 26 asymptomatic women (50 breasts; median age 33 years) with Baker I (15 breasts) or Baker II (35 breasts) capsules after aesthetic augmentation with round textured silicone implants in a subglandular or dual-plane pocket, and a study group of 25 women (25 breasts; median age 52 years) with surgically confirmed Baker III/IV contracture after post-mastectomy reconstruction with silicone implants (smooth or textured) in a subpectoral or prepectoral position. Patients with prior breast irradiation, prior revision surgery for CC, implant rupture, active infection or autoimmune disease were excluded from both groups. Two-dimensional SWE was performed by a single senior operator on a SuperSonic MACH 30 system with an L18-5 linear transducer in ShearWave PLUS mode (scale 0–300 kPa), with generous gel and minimal transducer pressure to limit pre-compression; a 2–4 mm ROI was placed in the stiffest region of the periprosthetic perimeter, and the mean, median, minimum, maximum and standard deviation of stiffness (kPa) were recorded as the median of three sequential acquisitions per position. Controls were examined at the 12 o’clock position only, whereas the study group was examined in four quadrants.

Median periprosthetic stiffness was 32.6 kPa in the control group and 138.0 kPa in the study group for the mean-stiffness parameter (32.5 and 138.0 kPa for the median-stiffness parameter), and all four primary stiffness parameters differed between the groups (*p* < 10^−11^). The areas under the receiver operating characteristic curves ranged from 0.988 to 0.994; for the median stiffness the area was 0.994 (95% CI 0.980–1.000), and a threshold of 82 kPa identified Baker III/IV contracture with 100% sensitivity, 98% specificity and 100% negative predictive value; the single false-positive result was an asymptomatic Baker II breast with a median stiffness of 121.5 kPa. Within the study group, median stiffness was higher in Baker IV (*n* = 15; 146.0 kPa, interquartile range 144.8–158.8; approximately 147 kPa in the abstract) than in Baker III (*n* = 10; 92.0 kPa, interquartile range 82.0–101.9) breasts (*p* = 0.0001). Within the control group, by contrast, median stiffness was similar in Baker I (31.4 kPa) and Baker II (33.1 kPa) breasts. The authors also note that a large proportion of Baker IV breasts, and a minority of Baker III breasts, reached or approached the 300 kPa ceiling of the device in at least one quadrant, so that the values reported for the most severe contractures are censored underestimates. Implant depth, periprosthetic tissue thickness, ROI diameter and body mass index did not influence stiffness within the groups, and in a multivariable logistic regression the median stiffness remained the only significant predictor of group membership (adjusted odds ratio 1.18 per kPa; 95% CI 1.08–1.31; *p* < 0.001) [25] (Figure 3).

These results show a large difference in periprosthetic stiffness between the two groups, but they cannot be read as an estimate of the diagnostic accuracy of SWE for contracture. The control and study groups differed not only in Baker grade but also in surgical indication (augmentation versus reconstruction), age (median 33 versus 52 years), body mass index, implant depth, periprosthetic tissue thickness (median 1.3 versus 3.1 mm), implant surface and pocket plane, and examination protocol (one versus four quadrants); the authors acknowledge this asymmetry and the incomplete documentation of implant characteristics in their report [25]. The examinations were, in addition, performed by a single operator, who placed the ROI in the stiffest part of the periprosthetic perimeter and whose blinding to the clinical group is not reported, and the threshold was derived and tested in the same cohort. Because patients with prior breast irradiation were excluded, the study does not inform on irradiated capsules, in which CC is most frequent [6,7], and its threshold cannot be assumed to apply to them. The near-perfect discrimination therefore reflects, to an extent that cannot be determined from the published data, the comparison of two separately recruited populations at opposite ends of the clinical spectrum—a design known to overestimate diagnostic performance (see Section 8); it does not show how SWE would perform in consecutive patients in whom contracture is suspected, and the 82 kPa threshold is a derivation-cohort value that has not been validated externally. It also differs from the cut-off of approximately 52 kPa suggested on a different platform in a reconstruction-only cohort [27].

### 4.6. Synthesis

Across the primary studies, three findings emerge. First, wherever periprosthetic stiffness was compared across Baker grades in cohorts that included high-grade contracture, higher grades were associated with higher stiffness or higher elastography scores [23,25,26,27,28], although Baker I and Baker II control breasts did not differ in the comparative study [25]. Second, in the one direct comparison, SWE was more reproducible than strain elastography and correlated more closely with the Baker score [27]. Third, in the one study that reported receiver operating characteristic analysis, stiffness separated Baker III/IV from Baker I/II breasts almost completely [25]; because the two groups also differed in surgical indication, age, tissue cover and examination protocol, this result shows that the groups were mechanically very different but does not establish the diagnostic accuracy of SWE for contracture (see Section 4.5). Against this, the relationship between stiffness and grade was described as limited in the abstract of the polyurethane implant report, although its Results give correlation coefficients of 0.75–0.87 [28], and the longitudinal augmentation series found no significant relationship between capsular elasticity and the Baker scale at follow-up [16] or between preoperative stiffness and the 12-month Baker grade [24], which may reflect the low prevalence of high-grade contracture expected within the first one to two years after primary augmentation [3,4,5].

The heterogeneity of the studies is considerable. The measurement target ranged from the capsule or periprosthetic perimeter [16,25,26,27] to the surrounding fat, gland, fascia and muscle sampled in four quadrants [15,24]; the parameter was reported as Young’s modulus in kPa [15,16,24,25,26,27], as shear-wave speed in m/s [28] or as a semi-quantitative score [23]; the devices differed (Aixplorer [16,24,26], GE LOGIQ E9 [27,28], SuperSonic MACH 30 [25], Siemens Acuson Antares Premium [23] and Siemens Acuson S3000 [28]); and the reference standard was the original or the modified Baker classification assessed by a variable number of examiners, with surgical confirmation in only one study [25]. No study reported the histological composition of the capsules examined. Absolute values are correspondingly disparate: contracted capsules measured 17.6–27.3 kPa on an Aixplorer system [26], the cut-off suggested on a GE LOGIQ E9 system was approximately 52 kPa [27], and on a SuperSonic MACH 30 system the derived threshold was 82 kPa, with median values of 92 and 146 kPa in Baker III and IV breasts [25]. For these reasons a quantitative pooling of results is not possible, and no cross-device stiffness threshold can be recommended at present.

## 5. Evidence on Shear-Wave Elastography for Predicting the Occurrence of Capsular Contracture

### 5.1. Early Postoperative Stiffness Dynamics

The Poznań group was the first to measure periprosthetic stiffness serially in the early postoperative period. In 19 women (38 breasts) aged 20–41 years undergoing bilateral dual-plane augmentation with textured silicone implants (180–545 mL), SWE and applanation tonometry were performed before surgery and on postoperative days 7 and 14, with daily pain scoring on a visual analogue scale [15]. The stiffness of all breast tissues changed significantly after surgery, peaking on day 7 and decreasing by day 14; the mean stiffness of the periprosthetic capsule, which was measurable only after surgery, was 20.6–27.7 kPa across the four quadrants on day 7 and 18.2–22.1 kPa on day 14. Pain correlated significantly with the stiffness of the lower-quadrant periprosthetic tissue between postoperative days 4 and 10 (Spearman coefficients 0.42–0.63) and with upper-quadrant glandular stiffness on days 6–10, whereas the stiffness of fat and of the thoracic fascia did not correlate with pain and that of muscle only on day 6; implant volume correlated, inversely, with pain only in the first postoperative days and with no SWE parameter, and applanation tonometry did not correlate with any parameter [15]. The authors interpreted early periprosthetic stiffening as an expression of the inflammatory reaction to the implant. A case report from the same group illustrated plane color-coded stiffness mapping of contracted capsules, compared with applanation tonometry, before and after secondary surgery in two patients with Baker III–IV contracture 6–7 months after submuscular augmentation [26]. Capsule stiffness, measured on an Aixplorer system as the mean of four quadrants, was 17.6 and 27.3 kPa in the two contracted breasts and 12.5 and 15.3 kPa in the contralateral Baker I breasts; ten days after capsulectomy it was 22.4 kPa in the breast that also received a larger implant and 20.5 kPa in the other, and no problems with shear-wave penetration were encountered [26].

### 5.2. Longitudinal Follow-Up Beyond the First Year

In a subsequent follow-up study of 13 patients (26 breasts) operated on during the same period (September 2010 to March 2011) as the early postoperative series [15], after dual-plane augmentation with textured silicone implants, SWE (Aixplorer, SuperSonic Imagine) and tonometry were repeated before surgery, on days 7 and 14 and after 8.5–21 months (mean 14.4 months) [16]. This report and the early postoperative series [15] are unlikely to be independent. Both come from the same plastic surgery practice and concern women operated on by a senior plastic surgeon between September 2010 and March 2011, with the same age range (20–41 years), implant type and dual-plane technique and the same preoperative, day 7 and day 14 examinations; the later report is presented as a follow-up study and cites the earlier one for its methods, but it describes its 13 patients as volunteers recruited from 200 consecutive augmentations and does not state whether they were among the 19 patients examined earlier [15,16]. That they were is the most plausible reading, and the two reports are therefore counted as one population when the number of individual patients is estimated (Section 2.3). The two patients of the case report from the same group, which was submitted in November 2010 and describes contracture six to seven months after augmentation [26], were necessarily operated on before this period. Mean capsular stiffness decreased from 19.6–25.0 kPa across the four quadrants on day 7 to 15.0–19.5 kPa at the final visit and did not correlate with capsule thickness on harmonic imaging. Tissue elasticity differed significantly between the preoperative examination and the first postoperative week (*p* < 0.05) but was not significantly different from baseline by the end of follow-up; the greatest decrease in pericapsular elasticity was observed in the lower and inner quadrants; and no significant relationship was found between capsular elasticity and the Baker scale (*p* > 0.05; the correlation for the inner quadrants at the final visit was borderline, R = 0.38, *p* = 0.058) or between the duration of follow-up and the change in tissue elasticity [16]; however, no breast exceeded a modified Baker score of 2.5, so that high-grade contracture could not be examined. The authors nevertheless considered sonoelastography a precise method for evaluating capsule formation that is capable of showing changes not detectable by palpation.

### 5.3. A Prospective 12-Month Study of Elastography and Tonometry as Predictive Tools

Kubasik and colleagues reported the only study explicitly designed to test SWE and tonometry as predictive tools [24]. Thirty-five women undergoing bilateral primary augmentation with anatomical textured silicone implants (Allergan and Eurosilicone; mean volume 329.0 ± 71.3 mL) in a dual-plane pocket were enrolled, and 28 (56 breasts; mean age 31 ± 5.0 years) completed the follow-up. Examinations were performed before surgery, on postoperative days 7 and 14, and after at least 12 months (mean 14.4 months; range 12–24 months). SWE was performed on an Aixplorer system (SuperSonic Imagine) with a linear transducer and a 2 mm ROI at the tissue–implant interface; adipose, glandular, muscular and fascial tissue and the peri-implant zone were measured in four quadrants. The surgeon who performed tonometry and palpation was blinded to the ultrasound and elastography results of the sonographer, and contracture was graded at the final visit with the modified Baker classification (IA = 1.0, IB = 1.5), a capsule thickness above 2 mm on harmonic imaging being classified as Baker III/IV; the distribution of Baker grades at the final visit was not reported. There was no control group, which the authors justified on the basis of the stability of breast tissue elasticity over time in their earlier menstrual-cycle study [34]. The examination schedule and the mean follow-up (14.4 months) are identical to those of the earlier follow-up report from the same group [16], and the early postoperative stiffness values are similar; however, the recruitment period is not stated, the ethics approval dates from January 2016, whereas the earlier series was operated on in 2010–2011 [15,16], and the incision (lower areolar versus inframammary fold) and the implant manufacturers differ, so that an overlap between the cohorts can be neither confirmed nor excluded from the published information.

All tissues were stiffer on days 7 and 14 than before surgery and most remained stiffer at 12 months, although the 12-month values were significantly lower than the early postoperative values. Preoperative tonometry predicted postoperative tonometry (Pearson r = 0.675, 0.656 and 0.657 at the three postoperative visits; all *p* < 0.001), but tonometry did not correlate with the Baker grade, and neither did preoperative SWE. Of the 48 correlations tabulated between SWE values in specific tissues and quadrants at day 7, day 14 or 12 months and the 12-month Baker score, five were marked as statistically significant (Spearman coefficients 0.28–0.46), and no adjustment for multiple comparisons was reported; only one of the five concerned the peri-implant region (inner quadrant at 12 months; coefficient 0.33, *p* = 0.047), and its reported 95% CI (−0.013 to 0.667), like that of one other, included zero [24]; the authors themselves described these correlations as weak associations rather than validated predictive relationships, and Iliescu-Glaja and colleagues likewise characterized them as modest [25]. The authors concluded that SWE is a precise and objective tool for detecting capsular changes that may improve the early prediction of contracture, but that, at present, elastography and tonometry are suited to monitoring during follow-up rather than to prediction.

### 5.4. Synthesis

Taken together, the longitudinal data show that SWE can track the dynamic stiffening and subsequent partial normalization of the periprosthetic tissues during the first year after augmentation, and that early stiffness reflects inflammation and pain rather than the eventual capsule phenotype. No study has yet demonstrated that an early SWE measurement identifies, with clinically useful accuracy, the breasts that will later develop Baker III/IV contracture; the predictive value of SWE therefore remains unproven. Notably, the only registered trial we identified that uses SWE as an outcome measure—the PREPER randomized trial of prepectoral versus subpectoral implant-based reconstruction in patients receiving PMRT—uses two-dimensional SWE together with the Baker classification as coprimary outcomes for grade ≥3 contracture at 6 months [19], indicating that the technique is beginning to be adopted as a trial endpoint before its validation.

## 6. Modifiers of Capsule Biology: Indication, Radiotherapy, Implant Surface and Pocket Plane

The populations in which SWE has been studied differ in ways that matter for capsule mechanics. Reconstruction after mastectomy removes the glandular layer, places the implant beneath thin skin flaps or muscle and is often followed by radiotherapy, which increases the odds of CC several-fold [6,7]; augmentation preserves the gland and is rarely irradiated. Of the eight reports, one concerns reconstruction patients only [27], one compares augmentation controls with reconstruction cases [25], one concerns revision surgery for prior contracture after either indication [28], one combines augmentation and reconstruction [23] and four concern primary augmentation [15,16,24,26]. No SWE study has stratified its results by radiotherapy exposure, and none has compared irradiated with non-irradiated capsules; both reconstruction cohorts examined with two-dimensional SWE excluded previously irradiated patients [25,27], and radiotherapy exposure is not reported in the other studies that included reconstruction patients [23,28]. Radiotherapy may also change the stiffness of the tissues that share the ROI with the capsule, independently of the capsule itself, but the available evidence, which comes from breasts without implants, is limited and not uniform. In a prospective study of 41 women irradiated after breast-conserving surgery, the maximum SWE elasticity of irradiated breast tissue outside the tumor bed did not increase: it fell from 19.4 ± 9.8 kPa before radiotherapy to 15.1 ± 7.0 kPa at 12 months, in parallel with the non-irradiated contralateral breast (20.3 ± 10.0 to 14.7 ± 6.3 kPa), whereas tumor-bed elasticity at 12 months was higher in patients with higher clinical grades of fibrosis [36]. In a small prospective case series, by contrast, the SWE stiffness of the pectoralis major, the muscle that covers a subpectoral implant, increased from one month after breast radiotherapy while its thickness decreased [37]. The direction and size of any effect of radiotherapy on periprosthetic SWE values are therefore unknown, and neither the values nor the thresholds obtained in non-irradiated cohorts [25,27] can be assumed to apply to irradiated breasts.

Implant surface and pocket plane are established modifiers of CC risk, although the direction and magnitude of their effects differ between settings. In augmentation, meta-analyses found lower CC rates with textured than with smooth implants, particularly in the subglandular position [38,39], and a meta-analysis of implant location reported a lower risk with submuscular than with subglandular placement [40]. In reconstruction, a 2024 systematic review and meta-analysis found no significant difference in CC between prepectoral and subpectoral placement (odds ratio 1.21; 95% CI 0.75–1.95) and no significant difference between smooth and textured implants, whereas saline implants were associated with less CC than silicone implants [41]. Within the SWE literature, implant characteristics were homogeneous within a study (textured anatomical implants in a dual-plane pocket [24]; smooth implants [23]; polyurethane-coated implants [28]), heterogeneous and incompletely documented [25] or described only as standard silicone implants [27], so that the effect of surface and plane on periprosthetic stiffness is unknown.

At the tissue level, capsule thickness and collagen organization increase with contracture, but not linearly: in a histological series, capsule thickness differed significantly between Baker I and higher grades but not among grades II–IV [42], whereas ultrasound-measured thickness increased from Baker grade II to grade IV [31]. The relationship between elastographic stiffness and the histological features of the capsule (thickness, collagen density and alignment, myofibroblast content) has not been examined in any SWE study and represents a key gap.

## 7. Comparison with Other Objective Methods

Table 4 compares SWE with the other objective methods that have been proposed for CC. Applanation tonometry [43,44], mammary compliance [45] and the breast durometer [46] measure whole-breast compressibility or surface firmness rather than the capsule itself; in the studies that performed tonometry and SWE simultaneously, tonometry did not correlate with pain, with the SWE parameters, with capsule thickness or with the Baker grade [15,16,24]. Capsule thickness on B-mode ultrasound increases with the Baker grade [31], but the systematic review by Mohan and colleagues found this relationship to be inconsistent across studies [2]. MRI provides anatomical detail of the capsule and implant, has been used to refine the clinical classification [47] and to visualize implant deformation in three dimensions in patients with contracture [48], and is reviewed elsewhere together with ultrasound [49], but it is costly and less accessible. Histology remains the reference for capsule structure but requires capsulectomy [2,42]. Its diagnostic performance against the Baker grade has been quantified in a large series: Larsen and colleagues developed procedure-specific histopathological scoring systems from 720 capsule biopsies of 542 patients undergoing implant exchange or removal, with the preoperative Baker grade (III/IV versus I/II) as the reference standard [50]. The augmentation score (collagen layer thickness, fiber organization, inflammatory infiltration and calcification) had an area under the receiver operating characteristic curve (AUC) of 0.81 on internal validation and of 0.84 (sensitivity 82%, specificity 75%) in a held-out test set; the reconstruction score (fiber organization, cellularity of the collagen layer and inflammatory infiltration) had an AUC of 0.72 on internal validation but of only 0.56 (sensitivity 33%, specificity 86%) in the test set, which the authors attributed to the small and heterogeneous reconstruction sample. As those authors point out, the limited interobserver agreement of the Baker grade constrains the accuracy that any index test can reach against it [50], which is a further reason not to read the AUC of 0.994 obtained for SWE in a two-group comparison [25] as an estimate of diagnostic accuracy (see Section 4.5). Strain elastography is semiquantitative and operator-dependent [23,27]. Compared with these methods, SWE is quantitative, does not require compression, is available on most modern ultrasound systems and can be repeated at the bedside; its disadvantages are device dependence, the physical constraints of a thin layer next to an implant (see Section 3.2), the absence of a validated threshold and the limited depth of penetration.

## 8. Limitations of the Current Evidence and Requirements for Standardization

The limitations of the evidence base are substantial and should temper enthusiasm for immediate clinical adoption:

- **Sample size and design:** The largest studies included 51 patients in a comparative design [25] and 28 patients in a prospective cohort [24]; the others included between 2 and 20 patients. No study was multicenter, none was powered on a prespecified hypothesis about diagnostic accuracy or prediction, and none included an independent validation cohort. Because the two earliest Poznań series probably share patients (Section 2.3 and Section 5.2), the eight reports also represent fewer independent observations than their combined sample size suggests.
- **Spectrum and confounding**: The only study that reported diagnostic accuracy estimates compared soft capsules after augmentation with severe contracture after reconstruction [25]; intermediate grades, reconstruction patients without contracture, augmentation patients with contracture and irradiated breasts were not represented, so that the reported accuracy cannot be transferred to a consecutive clinical population. In an empirical analysis of 184 studies evaluating 218 diagnostic tests, studies that compared a diseased group with a separate control group overestimated diagnostic performance relative to studies of a clinical population (relative diagnostic odds ratio 3.0; 95% CI 2.0–4.5) [51].
- **Reference standard**: All studies relied on the Baker classification, whose interobserver reliability is poor [10]; surgical confirmation was available in one study [25] and histology in none. Procedure-specific histopathological scoring systems are now available as a complementary reference; the score for augmentation capsules was reproduced in a test set, whereas that for reconstruction capsules was not [50].
- **Technical heterogeneity**: Measurement sites, ROI sizes, parameters (kPa, ARFI values, scores), devices and the number of acquisitions differed between studies; measures to limit precompression were described in a single study [25], and the rate of failed acquisitions in none (three studies excluded breasts or images of inadequate quality without stating their number [25,27,28]); reproducibility was quantified in only two studies, as the interobserver agreement of a strain elastography score (kappa 0.83) [23] and as the same-operator repeatability of SWE (ICC 0.878) [27], so that the interobserver reproducibility of periprosthetic SWE has not been assessed.
- **Specificity of the measurement:** Because the ROI (1–4 mm; a fixed 10 mm window for ARFI [28]) is usually larger than the capsule, the value obtained is a composite of the capsule and the adjacent periprosthetic tissues (Section 3.2). It can therefore be influenced by factors unrelated to contracture, among them transducer precompression [30], early postoperative inflammation [15,16,24], the age-related stiffness of the native tissues [33], the stiffness of the implant gel, which increases with implantation time [18], and possibly previous radiotherapy (Section 6). A given value must be interpreted in the light of these factors, which neither of the proposed thresholds takes into account.
- **Publication and optimism bias**: The literature consists of small, single-center, largely positive studies, and near-perfect accuracy estimates from derivation cohorts are prone to overfitting.

Because no clinical guideline from the ultrasound or plastic surgery societies currently addresses elastography of the implant capsule, we propose in Table 5 a minimum set of acquisition and reporting items, derived from the WFUMB and EFSUMB recommendations for breast elastography [13,14,22] and from the methodological gaps identified in the included studies. Adoption of such a standard would allow future studies to be compared and pooled.

## 9. Future Directions

The central unmet need is a properly designed predictive study (Figure 4). Such a study should enroll consecutive patients from both augmentation and reconstruction cohorts, record the implant and treatment characteristics that modify capsule biology, perform SWE with a standardized protocol before surgery, in the early postoperative period and at 6, 12 and 24 months, and grade contracture at each visit with two blinded raters. Preoperative SWE of the native tissues is feasible in such a design: in 60 breasts of 30 women, preoperative tissue stiffness measured with SWE was used to predict the expansion of the lower pole of the breast after augmentation with smooth implants over 24 months of follow-up [52]. Given expected rates of Baker III/IV contracture of roughly 5–25% at one to two years depending on the setting [3,4,5,7], several hundred breasts would be needed to observe a sufficient number of events for a multivariable time-to-event analysis; for example, approximately 300 breasts would be required to observe 30 events at an incidence of 10%. Early stiffness values should be analyzed as predictors of subsequent contracture, with hazard ratios adjusted for radiotherapy, surface, plane and indication, and any threshold derived should be tested in a separate validation cohort.

Five further priorities follow from the gaps identified above. First, the 82 kPa threshold proposed by Iliescu-Glaja and colleagues [25], and the cut-off of approximately 52 kPa suggested by Sowa and colleagues [27] should be validated in indication-matched cohorts (augmentation-only and reconstruction-only) of consecutive patients that include the full spectrum of Baker grades. Second, radiotherapy-stratified studies are needed, because the effect of radiotherapy on periprosthetic SWE values is unknown (see Section 6); the PREPER trial [19] provides a model in which SWE is measured within a randomized design. Third, stiffness measured before capsulectomy should be correlated with the histology of the excised capsule, which would anchor the elastographic signal to its biological substrate; the histopathological scoring systems of Larsen and colleagues offer a standardized reference for this purpose, with the reservation that only the augmentation score was reproduced in a test set, and those authors likewise proposed linking the score to preoperative ultrasound findings [50]. Fourth, phantom and simulation studies should quantify the thin-layer and interface effects described in Section 3.2 and establish cross-device calibration, as recommended in the general elastography guidelines [13,22]. Such work is feasible: in a standardized animal (chicken-breast) model, shear-wave measurements on the implant itself did not distinguish intact from ruptured devices, whereas strain elastography patterns did, indicating that quantitative SWE is unreliable within the implant [53]. Fifth, patient-reported outcomes should be included, because the clinical relevance of contracture ultimately lies in symptoms and appearance rather than in a stiffness value. Stiffness and symptoms can dissociate: in a recent cross-sectional study, the glandular parenchyma of breasts with subpectoral silicone implants was stiffer than that of non-augmented breasts more than one year after surgery, without any relationship to chronic mastalgia [54].

Finally, the clinical role that SWE could eventually play should be defined realistically. Even a validated threshold would not, by itself, establish the indication for capsulectomy or implant exchange. The clinical grades that define significant contracture are built on symptoms and appearance (pain, distortion, an unacceptable result; see Table 1) [8,9], which a stiffness value does not capture, and stiffness and symptoms can dissociate [54]: the single control breast that exceeded the 82 kPa threshold in the comparative study (121.5 kPa) was asymptomatic, and the authors recommended closer clinical surveillance for breasts that exceed the threshold without clinical features of contracture and described the threshold as an adjunct to clinical assessment [25]. Revision surgery is, moreover, undertaken for reasons other than contracture: in the series of 542 patients from which the histopathological scores were derived, contracture was the indication for implant exchange or removal in fewer than half of the patients after either augmentation or reconstruction, the others being operated on for cosmetic reasons, implant age or suspected rupture, among other indications [50]. Implant integrity, in particular, cannot be judged with SWE, which did not distinguish intact from ruptured devices in an experimental model [53], and is assessed with conventional ultrasound and MRI [49]. Because the measurement is not specific to the capsule (Section 3.2 and Section 8), an isolated high value may also reflect the adjacent tissues, the time since surgery or previous treatment rather than contracture. SWE is therefore best regarded as an adjunctive objective measurement, and the aim of future work should be less a single stiffness value that indicates surgery than a standardized assessment algorithm in which a protocol-based SWE measurement is integrated with the clinical grade, patient-reported symptoms and aesthetic concerns, implant integrity on imaging and the patient-, implant- and treatment-related factors listed in Table 5. Such an algorithm, which is in line with the recommendation that more than one modality be used to assess contracture [2], would itself have to be derived and validated prospectively (see Figure 4).

## 10. Conclusions

Shear-wave elastography is, at present, a promising objective adjunct to the Baker classification for assessing the severity of established capsular contracture. Across eight small studies from four research groups, two of which probably share patients, periprosthetic stiffness increased with the Baker grade in every cohort that included high-grade contracture; SWE was more reproducible and better correlated with the clinical grade than strain elastography after reconstruction; and in a comparative study it separated surgically confirmed Baker III/IV breasts after reconstruction from Baker I/II breasts after augmentation almost completely. Because that design confounds contracture with surgical indication, age, tissue cover and examination protocol, the result shows that the two groups were mechanically very different but is not evidence of the diagnostic accuracy of SWE. The evidence for predicting the future occurrence of contracture is preliminary: early postoperative stiffness reflects inflammation and pain, only modest associations with the 12-month grade have been reported, and no validated early predictor exists. The literature is limited by small single-center samples, heterogeneous techniques and reference standards, unreported technical failure rates and the absence of external validation (the two cut-offs proposed so far, approximately 52 kPa and 82 kPa, were derived on different platforms), so that no stiffness threshold can yet be recommended for clinical decision-making. Nor would a validated threshold suffice on its own: the measurement is not specific to the capsule and may be influenced by the adjacent tissues and, possibly, by previous radiotherapy, and the indication for capsulectomy or implant exchange also depends on symptoms, aesthetic concerns, implant integrity and other patient- and treatment-related factors. SWE should therefore be developed as one objective component of a standardized assessment algorithm that integrates it with clinical findings, not as a stand-alone criterion for surgery. Standardized acquisition and reporting, indication-matched validation of proposed thresholds in consecutive patients, histological anchoring, radiotherapy-stratified data and adequately powered prospective predictive cohorts are the necessary next steps before SWE can be adopted as a clinical or trial endpoint for capsular contracture.

## Figures and Tables

**Figure 1:**
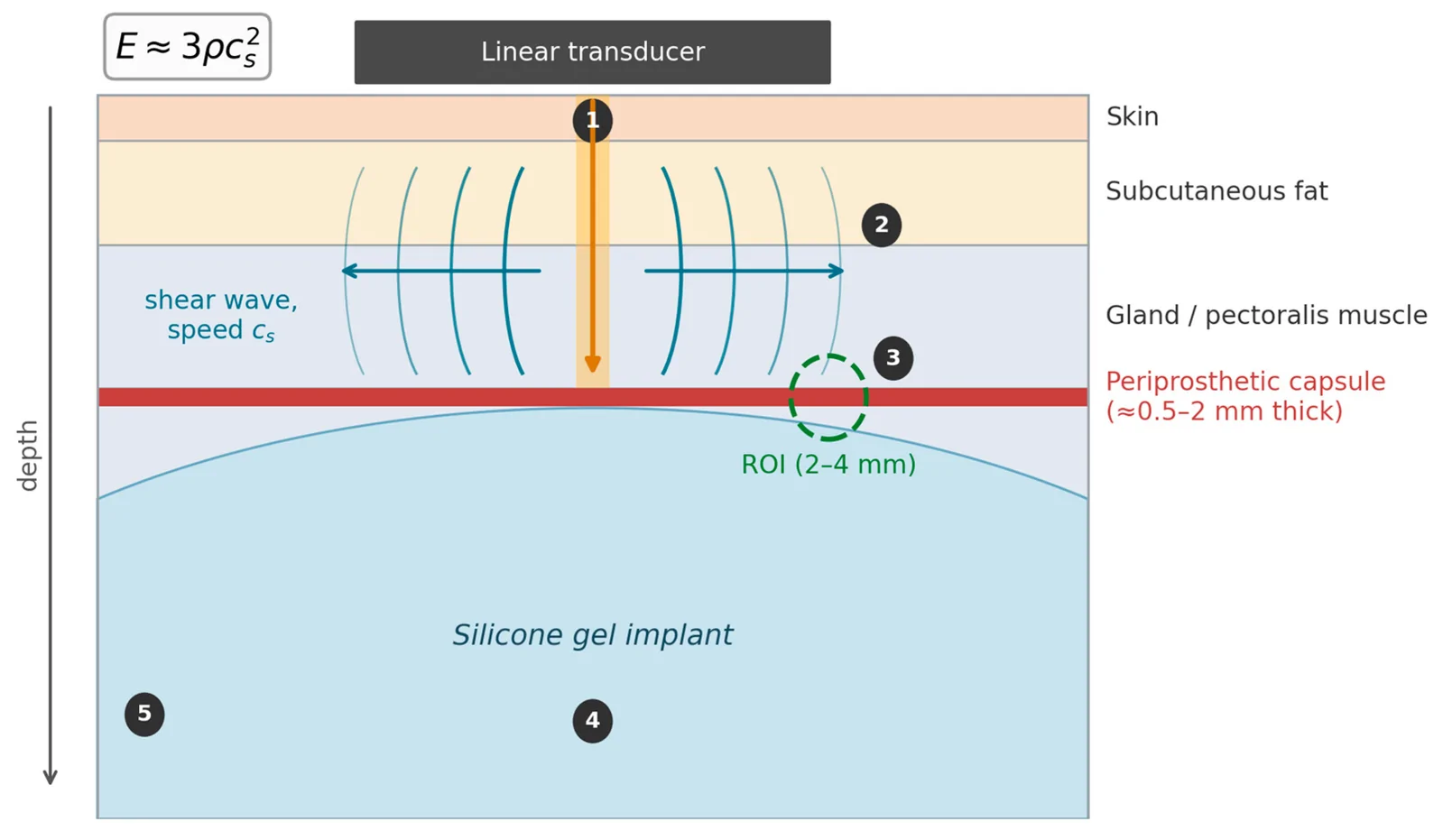
Schematic representation (not to scale) of shear-wave elastography at the tissue–implant interface. (1) A focused acoustic radiation force impulse displaces the tissue and (2) generates shear waves that propagate laterally; their speed c_s_ is tracked by ultrafast imaging and converted to a Young’s modulus (E ≈ 3ρc_s_^2^) under the assumptions of a linear, isotropic, homogeneous and incompressible medium [11,12,13]. (3) The periprosthetic capsule (≈0.5–2 mm [31]) is usually thinner than the region of interest used in published studies (1–4 mm [24,25,27]), so that most measurements average the capsule with adjacent tissue, and the thin-layer geometry may lead to dispersive guided-wave propagation [32]. (4) The silicone implant does not behave as soft tissue [17,18]. (5) Reliability of shear-wave tracking decreases with depth [13,21].

**Figure 2:**
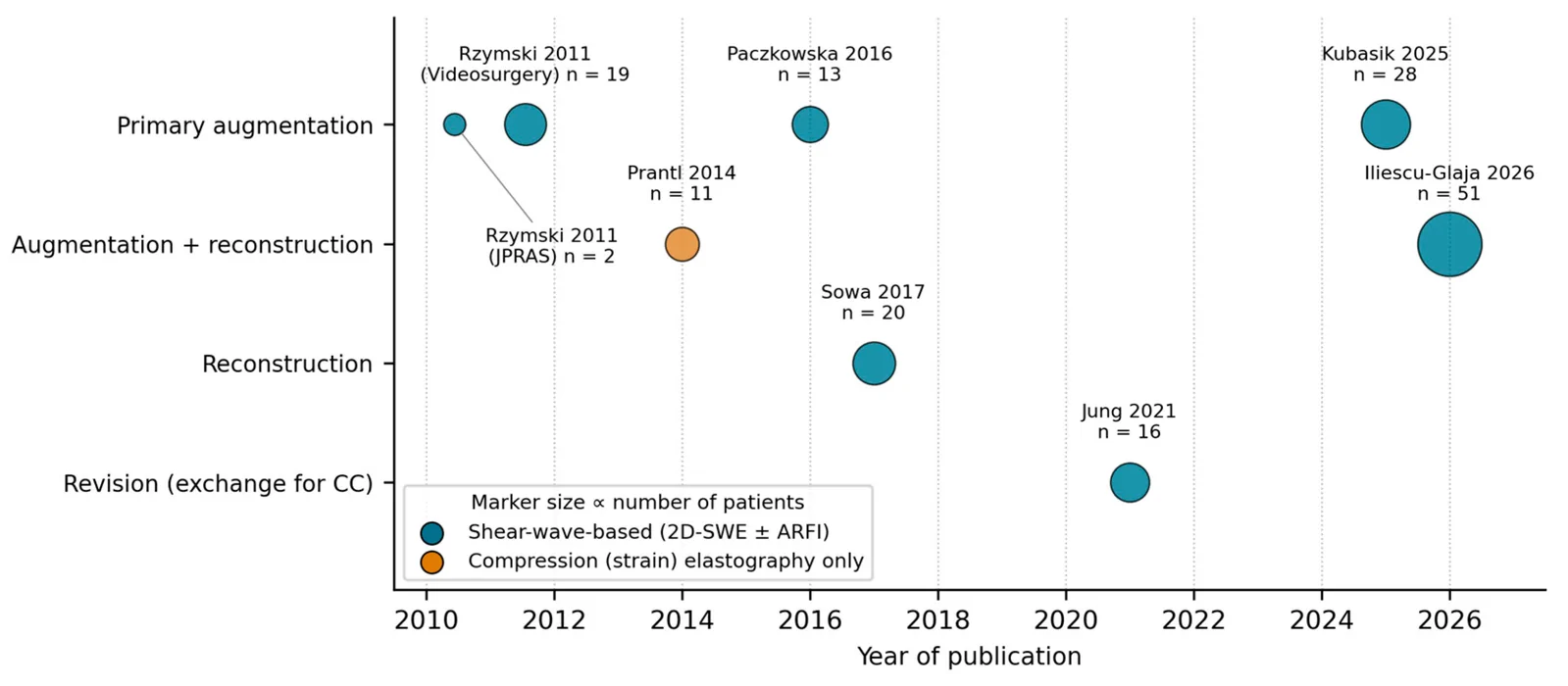
Evidence map of the primary clinical studies of elastography of the periprosthetic capsule (2011–2026), by year of publication and surgical indication. Marker size is proportional to the number of patients; color indicates the elastography technique. Data from Table 2 [15,16,23,24,25,26,27,28]. CC, capsular contracture; JPRAS, Journal of Plastic, Reconstructive & Aesthetic Surgery.

**Figure 3:**
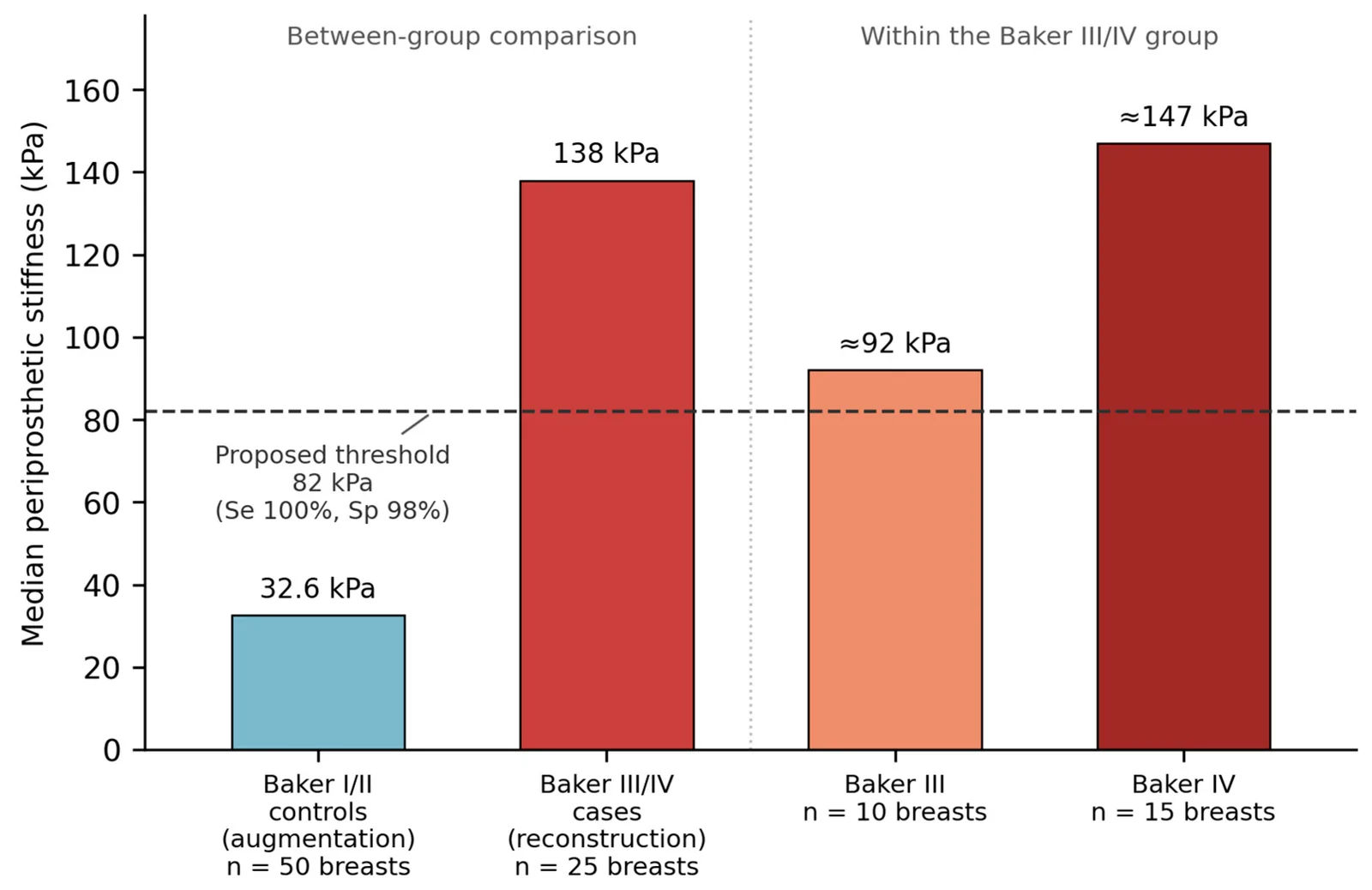
Median periprosthetic stiffness by Baker category as reported by Iliescu-Glaja et al. [25]. Left: control group (Baker I/II, aesthetic augmentation, 50 breasts) versus study group (surgically confirmed Baker III/IV, post-mastectomy reconstruction, 25 breasts). Right: Baker III and Baker IV breasts within the study group; these two values are reported as approximate (≈) by the authors in the abstract (92.0 and 146.0 kPa for the median-stiffness parameter in the full text). The dashed line indicates the 82 kPa threshold proposed in that study (sensitivity 100%, specificity 98%). Note that the two groups differ in surgical indication, implant characteristics and examination protocol, so that the difference cannot be attributed to contracture alone.

**Figure 4:**
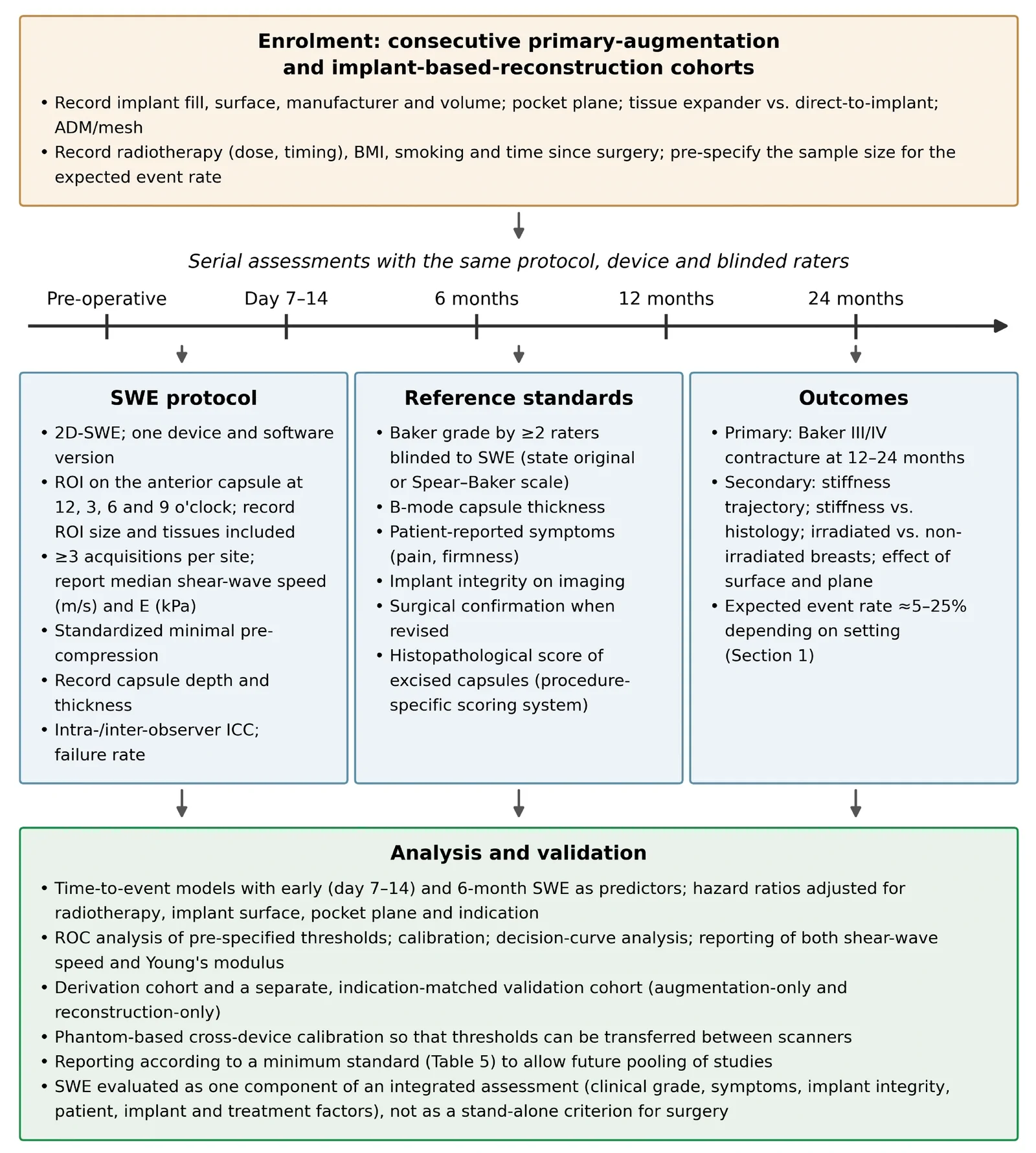
Proposed framework for a prospective study of shear-wave elastography as a predictor of capsular contracture, incorporating the acquisition and reporting standards of Table 5. The histopathological score listed among the reference standards refers to the procedure-specific scoring systems of Larsen et al. [50], of which the augmentation score, but not the reconstruction score, was reproduced in a test set; SWE is evaluated as one component of an integrated clinical assessment rather than as a stand-alone criterion for surgery. 2D-SWE, two-dimensional shear-wave elastography; ADM, acellular dermal matrix; BMI, body mass index; E, Young’s modulus; ICC, intraclass correlation coefficient; ROC, receiver operating characteristic; ROI, region of interest.

**Table 1:** The Baker classification of capsular contracture in its original form [8] and as modified by Spear and Baker for the reconstructed breast [9].

Grade	Original Baker Classification (Augmentation)	Spear–Baker Modification (Reconstruction)
I	Breast absolutely natural; no one can tell that the breast was augmented	IA: absolutely natural, cannot tell that the breast was reconstructed; IB: soft, but the implant can be detected by palpation or inspection because of limited soft-tissue cover
II	Minimal contracture; the surgeon can tell that surgery was performed, but the patient has no complaint	Mildly firm; implant visible or palpable
III	Moderate contracture; the patient feels some firmness	Moderately firm; implant readily detectable, but the result may still be acceptable
IV	Severe contracture; obvious on observation alone; the breast is hard, painful and distorted	Unacceptable result; breast firm, painful and/or distorted

**Table 2:** Primary clinical studies applying ultrasound elastography to the periprosthetic breast implant capsule (in chronological order).

Study, Country	Design	Patients/Breasts (Implants)	Indication and Implant Details	Elastography Technique and Device	Measurement Site and Parameter	Reference Standard/Endpoint
Rzymski 2011 [26], Poland	Case report	2/4 (2 contracted and 2 contralateral Baker I breasts)	Submuscular augmentation; Baker III–IV contracture 6–7 months after surgery; secondary surgery	SWE with plane color-coded stiffness maps, Aixplorer (SuperSonic Imagine), 15 MHz linear probe; applanation tonometry	Capsule, glandular and fatty tissue in four quadrants (mean of the quadrants); Young’s modulus (kPa)	Baker grade; before and 10 days after secondary surgery
Rzymski 2011 [15], Poland	Prospective, early postoperative	19/38	Primary bilateral augmentation; textured silicone implants (Natrelle, Allergan), 180–545 mL; dual-plane submuscular pocket; age 20–41 years	SWE (device not stated); applanation tonometry	Fat, gland, fascia, muscle and periprosthetic capsule by quadrant; Young’s modulus (kPa)	Daily VAS pain; pre-operative, day 7, day 14
Prantl 2014 [23], Germany	Prospective cross-sectional	11/17	7 bilateral aesthetic augmentations and unilateral reconstructions for asymmetry (4 according to the Abstract, 3 according to the Methods); smooth silicone gel implants, 125–325 cc, subpectoral; age 37–53 years; examined 1 week to 6 years after implantation	Color-coded compression (strain) elastography; Siemens Acuson Antares Premium, VFX13-5 (5–10 MHz); two radiologists blinded to the Baker grade	Stiff margin around the implant on the elastogram; four-grade score (1, no margin; 2, discontinuous margin; 3, continuous margin < 3 mm; 4, margin > 4 mm); B-mode score 0–5	Baker grade by two examiners in consensus (I: 6, II: 6, III: 3, IV: 2)
Paczkowska 2016 [16], Poland	Prospective longitudinal	13/26	Primary bilateral augmentation; textured silicone implants (Allergan); dual-plane pocket; age 20–41 years; same practice, surgical period and age range as [15], of which it is probably a subset (not stated; Section 5.2)	2D-SWE, Aixplorer (SuperSonic Imagine); applanation tonometry	Capsule/pericapsular tissue, fat, gland, fascia and muscle in four quadrants (fascia and muscle in the upper and inner quadrants only); Young’s modulus (kPa); capsule thickness on harmonic imaging at the last visit	Modified Baker scale (1, 1.5, 2, 2.5, 3, 4); surgeon and sonographer mutually blinded; preoperative, day 7, day 14, 8.5–21 months (mean 14.4)
Sowa 2017 [27], Japan	Comparative (strain vs. SWE)	20/27	Two-stage expander–implant breast reconstruction; age 24–68 years (mean 50.45); non-detectable capsule, previous breast surgery and previous radiotherapy excluded	Strain elastography (strain ratio) and SWE; GE LOGIQ E9, 9L linear probe (4–9 MHz); one operator, three measurements on the same day	Capsule just above the outer envelope of the implant; circular ROI 1–3 mm; mean elastic modulus (kPa)	Baker grade by two plastic surgeons (I: 6, II: 12, III: 5, IV: 4)
Jung 2021 [28], Germany	Follow-up after implant exchange	16/23 (abstract) or 25 (Methods)	Revision for prior contracture (initial implantation aesthetic in 7 and reconstructive in 9 patients); exchange to polyurethane-coated implants (Polytech); examined in the third year after exchange	Strain elastography (eight-level color score), SWE and ARFI quantification; Siemens Acuson S3000 (ARFI, 6–9 MHz transducer) and GE LOGIQ E9; single examiner blinded to the clinical findings	Glandular and peri-implant tissue in four quadrants and the subareolar region, three measurements each; shear-wave speed (m/s); fixed 10 mm ARFI window	Baker grade; VAS pain before and after exchange
Kubasik 2025 [24], Poland	Prospective longitudinal (predictive), no control group	28 (35 enrolled)/56	Primary bilateral augmentation; anatomical textured silicone implants (Allergan; Eurosilicone), mean 329.0 ± 71.3 mL; dual-plane pocket	2D-SWE, Aixplorer (SuperSonic Imagine), linear transducer; applanation tonometry	Adipose, glandular, muscular, fascial and peri-implant tissue in four quadrants (muscle and fascia in the upper and inner quadrants only); 2 mm ROI at the tissue–implant interface; kPa	Modified Baker grade (IA = 1.0, IB = 1.5) at ≥12 months (mean 14.4); capsule > 2 mm classed as III/IV; distribution of grades at follow-up not reported; pre-operative, day 7, day 14, ≥12 months
Iliescu-Glaja 2026 [25], Romania	Single-center comparative (STROBE); cases enrolled prospectively, control examinations acquired during routine follow-up and included retrospectively	51/75	Controls: 26 patients/50 breasts (median age 33 years), aesthetic augmentation, round textured silicone, subglandular or dual-plane, Baker I/II. Cases: 25 patients/25 breasts (median age 52 years), post-mastectomy reconstruction, silicone (smooth or textured), subpectoral or prepectoral, surgically confirmed Baker III/IV. Prior breast irradiation, prior revision for CC, implant rupture, active infection and autoimmune disease excluded in both groups	2D-SWE, SuperSonic MACH 30, L18-5 (5–18 MHz), ShearWave PLUS, 0–300 kPa; single senior operator; generous gel and minimal transducer pressure	Stiffest area of the periprosthetic perimeter; ROI 2–4 mm; mean, median, minimum, maximum, SD (kPa); median of three acquisitions per position; controls at 12 o’clock only, cases in four quadrants (averaged)	Baker I/II (one surgeon blinded to elastography) vs. III/IV (operating surgeon; surgical confirmation in all cases)

2D-SWE, two-dimensional shear-wave elastography; ARFI, acoustic radiation force impulse; ROI, region of interest; SD, standard deviation; SWE, shear-wave elastography; VAS, visual analogue scale.

**Table 3:** Principal quantitative results and main limitations of the primary studies.

Study	Relationship Between Elastography and the Baker Grade	Other Principal Findings	Main Limitations
Rzymski 2011 [26]	Capsule stiffness 17.6 kPa (Baker III/IV) and 27.3 kPa (Baker III) in the two contracted breasts vs. 12.5 and 15.3 kPa in the contralateral Baker I breasts	Ten days after capsulectomy: capsule 22.4 kPa (with exchange for a larger implant) and 20.5 kPa; tonometry 11% and 16% lower in the operated contracted breasts; no problems with shear-wave penetration	Two patients (four breasts); descriptive, without statistical analysis; blinding not reported; postoperative values obtained only 10 days after secondary surgery
Rzymski 2011 [15]	Not assessed (early postoperative period)	Stiffness of all tissues increased after surgery, highest on day 7, lower on day 14; periprosthetic capsule 20.6–27.7 kPa (by quadrant) on day 7 and 18.2–22.1 kPa on day 14; pain correlated with lower-quadrant periprosthetic stiffness (days 4–10; Spearman coefficients 0.42–0.63) and upper-quadrant glandular stiffness (days 6–10); no correlation for fat or fascia, and for muscle only on day 6; implant volume correlated inversely with pain only early and with no SWE parameter; tonometry correlated with no parameter; no problems with shear-wave penetration at the implant surface reported	No contracture endpoint (Baker grade not assessed) and follow-up limited to 14 days; 19 patients, no control group; elastography device not stated; numerous day- and quadrant-specific correlations without adjustment for multiple testing; population probably shared with [16]
Prantl 2014 [23]	Elastography score vs. Baker grade: Pearson r = 0.89; discordant in 4 of 17 implants (23.5%); the “kappa 0.83–0.89” of the abstract combines this coefficient with the interobserver kappa	Agreement between the two radiologists: Cohen’s kappa 0.83 (grades differed in 2 of 17 implants); B-mode score (0–5) also compared with the Baker grade	11 patients (17 implants), of which only five implants were Baker III/IV; semi-quantitative score devised by the authors, based on operator-applied compression (no absolute stiffness); implants examined from 1 week to 6 years after implantation; augmentation and reconstruction mixed; inconsistencies between abstract and full text
Paczkowska 2016 [16]	No significant relationship between capsular elasticity and the Baker scale (*p* > 0.05; borderline for the inner quadrants at the last visit, R = 0.38, *p* = 0.058); highest modified Baker score 2.5 (two breasts), i.e., no high-grade contracture	Significant change between preoperative values and day 7 (*p* < 0.05); no significant difference between preoperative values and the end of follow-up; mean capsular stiffness 19.6–25.0 kPa (by quadrant) on day 7 and 15.0–19.5 kPa at the last visit, with the greatest decrease in the lower and inner quadrants; capsular stiffness not correlated with capsule thickness on harmonic imaging; tonometry not correlated with capsular stiffness or thickness; no relationship between follow-up duration and change in elasticity	13 volunteers recruited from 200 consecutive augmentations; no high-grade contracture (highest modified Baker score 2.5), so that the relationship with Baker III/IV could not be tested; no control group; population probably shared with [15]; described as a pilot study by its authors
Sowa 2017 [27]	Baker score vs. SWE r = 0.8124; Baker score vs. strain elastography r = 0.6983; cut-off of approximately 52 kPa for Baker III/IV suggested from histograms (sensitivity and specificity not reported)	Same-operator reproducibility: ICC 0.878 (95% CI 0.785–0.937) for SWE vs. 0.724 (95% CI 0.552–0.851) for strain; interobserver variability not assessed; correlation between strain and SWE r = 0.6788	20 patients (27 implants, nine Baker III/IV); single operator with same-day repeated measurements only, interobserver variability not assessed; cut-off read from histograms, without sensitivity, specificity or validation; blinding of the operator to the Baker grade and time since reconstruction not reported; breasts with a non-detectable capsule and previously irradiated patients excluded
Jung 2021 [28]	In the Results, ARFI vs. Baker score R = 0.873 and SWE vs. Baker score R = 0.754; mean ARFI velocity 2.1, 2.3, 2.6 and 2.5 m/s in Baker I, II, III and IV breasts; the abstract states that elastography correlated only to a limited extent with the Baker findings	Pain 4.1 ± 2.8 before exchange vs. 1.7 ± 1.0 at follow-up (*p* = 0.002); ARFI vs. pain R = 0.648 (*p* = 0.05) in the Results (r = 0.873 in the Abstract); ARFI velocities > 2.3 m/s (threshold adopted from liver fibrosis) regarded as indicating fibrosis; glandular stiffness did not differ between breasts with and without implants	16 patients, all after implant exchange for contracture to polyurethane-coated implants, in which the authors consider Baker grading more difficult; single examiner, interobserver variability not assessed; fixed 10 mm ARFI window; fibrosis threshold of 2.3 m/s adopted from liver elastography; number of implants and correlation coefficients inconsistent between abstract and full text
Kubasik 2025 [24]	Preoperative SWE not correlated with the 12-month Baker grade; five of 48 tabulated tissue- and quadrant-specific correlations between day 7, day 14 or 12-month SWE values and the 12-month Baker score (Spearman coefficients 0.28–0.46; peri-implant region only in the inner quadrant at 12 months: 0.33, *p* = 0.047, 95% CI −0.013 to 0.667); distribution of Baker grades at follow-up not reported; tonometry not correlated with the Baker grade	All tissues stiffer at days 7 and 14 than preoperatively; most still stiffer at 12 months, but lower than at days 7–14; peri-implant stiffness 19.6–25.5 kPa (by quadrant) on day 7 and 13.4–17.7 kPa at ≥12 months; preoperative tonometry predicted postoperative tonometry (r = 0.675, 0.656, 0.657; *p* < 0.001); age not associated with the Baker grade (rs = 0.13)	28 of 35 enrolled patients analyzed; no control group; distribution of Baker grades at follow-up not reported; capsule thickness > 2 mm classed as Baker III/IV, so that the reference grade was not independent of imaging; 48 correlations tested without adjustment for multiple testing, of which five were significant and weak; overlap with [16] not excluded
Iliescu-Glaja 2026 [25]	Group median of mean stiffness 32.6 kPa (Baker I/II) vs. 138.0 kPa (Baker III/IV) (32.5 vs. 138.0 kPa for median stiffness), *p* < 10^−11^; AUC 0.988–0.994 (median stiffness: 0.994, 95% CI 0.980–1.000); 82 kPa threshold: sensitivity 100%, specificity 98%, NPV 100%; Baker III 92.0 kPa (interquartile range 82.0–101.9) vs. Baker IV 146.0 kPa (interquartile range 144.8–158.8) for median stiffness (*p* = 0.0001; ≈92 vs. ≈147 kPa in the abstract)	Implant depth, tissue thickness, ROI diameter and BMI did not influence stiffness within groups; median stiffness the only significant predictor in multivariable logistic regression (adjusted OR 1.18 per kPa; 95% CI 1.08–1.31; *p* < 0.001); median stiffness similar in Baker I (31.4 kPa) and Baker II (33.1 kPa) control breasts; single false-positive control a Baker II breast with median stiffness 121.5 kPa; groups also differed in implant depth (0.8 vs. 1.2 cm) and periprosthetic tissue thickness (1.3 vs. 3.1 mm); 300 kPa measurement ceiling reached or approached in a large proportion of Baker IV and a minority of Baker III breasts	Two separately recruited groups that differed in indication, age, BMI, implant depth, tissue thickness, implant surface and plane; controls included retrospectively and examined at one position, cases at four; single center and single operator, ROI placed in the stiffest area, blinding of the operator to the group not reported; intra- and interobserver reliability not assessed; threshold derived and tested in the same cohort; irradiated breasts excluded; 300 kPa ceiling reached in some Baker III/IV breasts

AUC, area under the receiver operating characteristic curve; BMI, body mass index; CI, confidence interval; ICC, intraclass correlation coefficient; NPV, negative predictive value; OR, odds ratio; r_s_, Spearman correlation coefficient. Values are reported exactly as published; “≈” denotes values given as approximate by the authors. Limitations are those stated by the authors of each study or identified in this review (Section 4, Section 5 and Section 8).

**Table 4:** Objective methods proposed for the assessment of capsular contracture, compared with shear-wave elastography.

Method	What Is Measured	Evidence of a Relationship with the Baker Grade	Main Limitations
Baker classification (clinical reference) [8,9]	Firmness, visibility, distortion and pain by palpation and inspection	Reference standard by definition	Interobserver reliability poor (weighted kappa 0.55; 48% exact agreement) [10]
Applanation tonometry/mammary compliance/durometer [43,44,45,46]	Whole-breast compressibility or surface firmness	Proposed as objective measures of breast firmness; in SWE studies tonometry did not correlate with pain, SWE parameters, capsule thickness or Baker grade [15,16,24]	Measures the whole breast, not the capsule; influenced by implant size, tissue cover and technique
B-mode ultrasound capsule thickness [31]	Thickness of the capsule (mm)	Thickness increased across Baker grades II, III and IV (0.58 ± 0.11, 1.07 ± 0.16 and 1.89 ± 0.55 mm; grade I not measurable) [31]; relationship inconsistent across studies [2]	Submillimeter measurement; operator- and probe-dependent; thickness is not stiffness
MRI [47,48,49]	Capsule thickness, implant shape and deformation, fluid	Used to refine clinical classification [47]; three-dimensional implant deformation in contracture [48]	Cost, availability, no direct mechanical measure
Compression (strain) elastography [23,27]	Relative strain under manual compression (color map, score, ratio)	Score correlated with Baker grade (r = 0.89; discordant in 4 of 17 implants; interobserver kappa 0.83) [23]; correlation weaker than SWE (r = 0.70 vs. 0.81) [27]	Semi-quantitative; operator-dependent; applied stress unknown
Shear-wave elastography (this review)	Shear-wave speed (m/s) and Young’s modulus (kPa) of periprosthetic tissue	r = 0.81 with Baker score [27]; AUC 0.994 for Baker III/IV reconstruction vs. Baker I/II augmentation breasts in a two-group comparison, not an estimate of diagnostic accuracy [25]; R = 0.75 (SWE) and 0.87 (ARFI) in one cohort, described as limited in its abstract [28]	Device dependence; thin-layer and interface effects; depth; no validated threshold (cut-offs proposed: ≈52 kPa [27], 82 kPa [25]); ROI usually larger than the capsule; no data in irradiated breasts; small studies
Histology [2,42,50]	Capsule thickness, collagen organization, cellular infiltrate; composite histopathological score [50]	Thickness differed between Baker I and higher grades but not among grades II–IV [42]; histopathological score vs. Baker III/IV (720 biopsies, 542 patients): after augmentation, AUC 0.81 (internal validation) and 0.84 (test set; sensitivity 82%, specificity 75%); after reconstruction, AUC 0.72 (internal validation) and 0.56 (test set; sensitivity 33%, specificity 86%) [50]	Requires capsulectomy; not applicable for monitoring; reconstruction score not reproduced in the test set; Baker grade as reference standard; mostly textured implants [50]

AUC, area under the receiver operating characteristic curve; MRI, magnetic resonance imaging; ROI, region of interest; SWE, shear-wave elastography.

**Table 5:** Proposed minimum acquisition and reporting items for studies of shear-wave elastography of the periprosthetic capsule (proposal of the authors, informed by the WFUMB and EFSUMB elastography guidelines [13,14,22] and by the gaps identified in the included studies).

Domain	Items to Standardize and Report
Equipment	Manufacturer, system, software version, transducer and frequency; elastography mode (2D-SWE or point SWE/ARFI); stiffness scale
Acquisition	Patient position; probe orientation; minimal and standardized precompression (e.g., generous gel, no visible tissue deformation); number of acquisitions per site (≥3) and the statistic reported (median); use of the quality/reliability map where available; operator blinded to the clinical grade
Region of interest	Size (mm); exact placement (on the capsule, at a stated distance from the implant surface); clock positions examined (12, 3, 6, 9 o’clock as a minimum); depth of the capsule from the skin; capsule thickness on B-mode; tissues included in the ROI
Parameters	Shear-wave speed (m/s) and Young’s modulus (kPa) both reported; mean and median with dispersion; per-site and per-breast summary
Reliability	Intra- and inter-observer reliability (ICC with 95% CI); rate and causes of failed or unreliable acquisitions
Patient and implant	Indication (augmentation, reconstruction, revision); implant fill, surface, manufacturer, volume and shape; pocket plane; tissue expander vs. direct-to-implant; ADM or mesh; time since implantation; BMI
Treatment	Radiotherapy (yes/no, dose, timing relative to implantation); chemotherapy; prior capsulotomy or capsulectomy
Reference standard	Baker classification used (original or Spear–Baker); number of raters and blinding to elastography; surgical and histological confirmation when available (standardized histopathological score [50]); patient-reported symptoms
Analysis	Prespecified hypotheses; handling of bilateral breasts (clustering); derivation and validation cohorts for any threshold; reporting of sensitivity, specificity and calibration; evaluation of SWE together with the clinical grade, symptoms and patient, implant and treatment factors rather than as a single cut-off

2D-SWE, two-dimensional shear-wave elastography; ADM, acellular dermal matrix; ARFI, acoustic radiation force impulse; BMI, body mass index; CI, confidence interval; EFSUMB, European Federation of Societies for Ultrasound in Medicine and Biology; ICC, intraclass correlation coefficient; WFUMB, World Federation for Ultrasound in Medicine and Biology.

## Data Availability

The original contributions presented in this study are included in the article. Further inquiries can be directed to the corresponding authors.

## Abbreviations

The following abbreviations are used in this manuscript:

2D-SWE	two-dimensional shear-wave elastography
ADM	acellular dermal matrix
ARFI	acoustic radiation force impulse
AUC	area under the receiver operating characteristic curve
BMI	body mass index
CC	capsular contracture
CI	confidence interval
EFSUMB	European Federation of Societies for Ultrasound in Medicine and Biology
ICC	intraclass correlation coefficient
MRI	magnetic resonance imaging
NPV	negative predictive value
OR	odds ratio
PMRT	post-mastectomy radiotherapy
ROI	region of interest
STROBE	Strengthening the Reporting of Observational Studies in Epidemiology
SWE	shear-wave elastography
VAS	visual analogue scale
WFUMB	World Federation for Ultrasound in Medicine and Biology

## References

[1] Headon H., Kasem A., Mokbel K. (2015). Capsular Contracture after Breast Augmentation: An Update for Clinical Practice. Arch. Plast. Surg..

[2] Mohan A.S., Sullivan J., Tenenbaum M.M., Broderick K.B., Myckatyn T.M. (2024). Toward a Consensus Approach for Assessing Capsular Contracture Severity and Progression: A Systematic Review. Plast. Reconstr. Surg..

[3] Spear S.L., Murphy D.K., Allergan Silicone Breast Implant U.S. Core Clinical Study Group (2014). Natrelle Round Silicone Breast Implants: Core Study Results at 10 Years. Plast. Reconstr. Surg..

[4] Caplin D.A., Calobrace M.B., Wixtrom R.N., Estes M.M., Canady J.W. (2021). MemoryGel Breast Implants: Final Safety and Efficacy Results after 10 Years of Follow-Up. Plast. Reconstr. Surg..

[5] Maxwell G.P., Van Natta B.W., Bengtson B.P., Murphy D.K. (2015). Ten-Year Results from the Natrelle 410 Anatomical Form-Stable Silicone Breast Implant Core Study. Aesthet. Surg. J..

[6] Pu Y., Mao T.-C., Zhang Y.-M., Wang S.-L., Fan D.-L. (2018). The Role of Postmastectomy Radiation Therapy in Patients with Immediate Prosthetic Breast Reconstruction: A Meta-Analysis. Medicine.

[7] Vinsensia M., Schaub R., Meixner E., Hoegen P., Arians N., Forster T., Hoeltgen L., Köhler C., Uzun-Lang K., Batista V. (2024). Incidence and Risk Assessment of Capsular Contracture in Breast Cancer Patients Following Post-Mastectomy Radiotherapy and Implant-Based Reconstruction. Cancers.

[8] Baker J.L., Owsley J.Q., Peterson R.A. (1978). Augmentation mammaplasty. Symposium on Aesthetic Surgery of the Breast.

[9] Spear S.L., Baker J.L. (1995). Classification of Capsular Contracture after Prosthetic Breast Reconstruction. Plast. Reconstr. Surg..

[10] de Bakker E., Rots M., Buncamper M.E., Niessen F.B., Smit J.M., Winters H.A.H., Özer M., de Vet H.C.W., Mullender M.G. (2020). The Baker Classification for Capsular Contracture in Breast Implant Surgery Is Unreliable as a Diagnostic Tool. Plast. Reconstr. Surg..

[11] Sarvazyan A.P., Rudenko O.V., Swanson S.D., Fowlkes J.B., Emelianov S.Y. (1998). Shear Wave Elasticity Imaging: A New Ultrasonic Technology of Medical Diagnostics. Ultrasound Med. Biol..

[12] Bercoff J., Tanter M., Fink M. (2004). Supersonic Shear Imaging: A New Technique for Soft Tissue Elasticity Mapping. IEEE Trans. Ultrason. Ferroelectr. Freq. Control.

[13] Shiina T., Nightingale K.R., Palmeri M.L., Hall T.J., Bamber J.C., Barr R.G., Castera L., Choi B.I., Chou Y.-H., Cosgrove D. (2015). WFUMB Guidelines and Recommendations for Clinical Use of Ultrasound Elastography: Part 1: Basic Principles and Terminology. Ultrasound Med. Biol..

[14] Barr R.G., Nakashima K., Amy D., Cosgrove D., Farrokh A., Schafer F., Bamber J.C., Castera L., Choi B.I., Chou Y.-H. (2015). WFUMB Guidelines and Recommendations for Clinical Use of Ultrasound Elastography: Part 2: Breast. Ultrasound Med. Biol..

[15] Rzymski P., Kubasik M., Gaca M., Opala T. (2011). Is the Shear Wave Sonographic Elastography Correlated with Pain after Breast Augmentation with Silicone Implants an Indication of Inflammatory Activity? A Preliminary Report. Wideochir Inne Tech. Maloinwazyjne.

[16] Paczkowska K., Rzymski P., Kubasik M., Opala T. (2016). Sonoelastography in the Evaluation of Capsule Formation after Breast Augmentation—Preliminary Results from a Follow-up Study. Arch. Med. Sci..

[17] Kuehlmann B., Prantl L., Michael Jung E. (2016). Imaging of Idle Breast Implants with Ultrasound-Strain Elastography- A First Experimental Study to Establish Criteria for Accurate Imaging of Idle Implants via Ultrasound-Strain Elastography. Clin. Hemorheol. Microcirc..

[18] Ruffenach L., Heintz D., Villette C., Cosentino C., Funfschilling D., Bodin F., Bahlouli N., Chatelin S. (2024). Ultrasonic Elastography for the Prevention of Breast Implant Rupture: Detection of an Increase with Stiffness over Implantation Time. J. Biomech..

[19] Jeong J. (2020). Prospective Randomized Control Study About the Effect of Postmastectomy Radiation Therapy on Capsular Contracture in Patients with Prepectoral or Subpectoral Implant-Based Breast Reconstruction: PREPER Trial; Clinical Trial Registration NCT04391296. NCT04391296.

[20] Nightingale K., Soo M.S., Nightingale R., Trahey G. (2002). Acoustic Radiation Force Impulse Imaging: In Vivo Demonstration of Clinical Feasibility. Ultrasound Med. Biol..

[21] Sigrist R.M.S., Liau J., Kaffas A.E., Chammas M.C., Willmann J.K. (2017). Ultrasound Elastography: Review of Techniques and Clinical Applications. Theranostics.

[22] Bamber J., Cosgrove D., Dietrich C.F., Fromageau J., Bojunga J., Calliada F., Cantisani V., Correas J.-M., D’Onofrio M., Drakonaki E.E. (2013). EFSUMB Guidelines and Recommendations on the Clinical Use of Ultrasound Elastography. Part 1: Basic Principles and Technology. Ultraschall Med..

[23] Prantl L., Englbrecht M.A., Schoeneich M., Kuehlmann B., Jung E.M., Kubale R. (2014). Semiquantitative Measurements of Capsular Contracture with Elastography--First Results in Correlation to Baker Score. Clin. Hemorheol. Microcirc..

[24] Kubasik M., Rzymska A., Pięta B., Rzymski P. (2025). Ultrasound Elastography and Tonometry as Predictive Tools for Capsular Contracture After Breast Implant Surgery: Over a 12-Month Prospective Study. J. Clin. Med..

[25] Iliescu-Glaja M., Simion F., Stoian D., Grujic D., Tarta C., Crainiceanu Z., Motoc A. (2026). Shear-Wave Elastography as an Objective Diagnostic Tool for Capsular Contracture After Breast Implant Surgery: A Comparative Study. Diagnostics.

[26] Rzymski P., Kubasik M., Opala T. (2011). Use of Shear Wave Sonoelastography in Capsular Contracture before and after Secondary Surgery: Report of Two Cases. J. Plast. Reconstr. Aesthet. Surg..

[27] Sowa Y., Yokota I., Itsukage S., Nakatsukasa K., Sakaguchi K., Taguchi T., Numajiri T. (2017). Evaluation of the Severity of Capsular Contracture Using Elastography after Breast Implant Reconstruction. Clin. Hemorheol. Microcirc..

[28] Jung E., Hösl V., von Fraunberg S., Jung F., Prantl L. (2021). Ultrasound Elastography for the Detection of Capsular Fibrosis in Breast Implants: First Results. Clin. Hemorheol. Microcirc..

[29] Cosgrove D.O., Berg W.A., Doré C.J., Skyba D.M., Henry J.-P., Gay J., Cohen-Bacrie C. (2012). BE1 Study Group. Shear Wave Elastography for Breast Masses Is Highly Reproducible. Eur. Radiol..

[30] Barr R.G., Zhang Z. (2012). Effects of Precompression on Elasticity Imaging of the Breast: Development of a Clinically Useful Semiquantitative Method of Precompression Assessment. J. Ultrasound Med..

[31] Kim J.H., Nam S.E., Sung J.Y., Song K.Y., Bang B.S., Lee E.K. (2022). The Value of Capsule Thickness on Breast Ultrasound as an Indicator of the Severity of Capsular Contracture and Its Correlation with the Baker Classification. Aesthetic Plast. Surg..

[32] Couade M., Pernot M., Prada C., Messas E., Emmerich J., Bruneval P., Criton A., Fink M., Tanter M. (2010). Quantitative Assessment of Arterial Wall Biomechanical Properties Using Shear Wave Imaging. Ultrasound Med. Biol..

[33] Rzymski P., Skórzewska A., Skibińska-Zielińska M., Opala T. (2011). Factors Influencing Breast Elasticity Measured by the Ultrasound Shear Wave Elastography—Preliminary Results. Arch. Med. Sci..

[34] Rzymski P., Skórzewska A., Opala T. (2011). Changes in Ultrasound Shear Wave Elastography Properties of Normal Breast during Menstrual Cycle. Clin. Exp. Obstet. Gynecol..

[35] Rzymski P.T., Wilczak M., Opala T. (2012). Influence of Sex Hormones in Women on Breast Elasticity Measured by Shear Wave Sonoelastography—A Cross-Sectional Study. Gynecol. Endocrinol..

[36] Jung W., Chung J., Lee J., Kim K. (2024). Quantifying Radiation-Induced Breast Fibrosis by Shear-Wave Elastography in Patients with Breast Cancer: A 12-Months-Follow-up Data of a Prospective Study. Clin. Transl. Radiat. Oncol..

[37] Wolfram S., Takayesu J.S.K., Pierce L.J., Jagsi R., Lipps D.B. (2023). Changes in Pectoralis Major Stiffness and Thickness Following Radiotherapy for Breast Cancer: A 12-Month Follow-up Case Series. Radiother. Oncol..

[38] Barnsley G.P., Sigurdson L.J., Barnsley S.E. (2006). Textured Surface Breast Implants in the Prevention of Capsular Contracture among Breast Augmentation Patients: A Meta-Analysis of Randomized Controlled Trials. Plast. Reconstr. Surg..

[39] Wong C.-H., Samuel M., Tan B.-K., Song C. (2006). Capsular Contracture in Subglandular Breast Augmentation with Textured versus Smooth Breast Implants: A Systematic Review. Plast. Reconstr. Surg..

[40] Egeberg A., Sørensen J.A. (2016). The Impact of Breast Implant Location on the Risk of Capsular Contraction. Ann. Plast. Surg..

[41] Christodoulou N., Secanho M., Kokosis G., Malgor R.D., Winocour J., Yu J.W., Mathes D.W., Kaoutzanis C. (2024). Capsular Contracture in Breast Reconstruction: A Systematic Review and Meta-Analysis. J. Plast. Reconstr. Aesthet. Surg..

[42] Bui J.M., Perry T., Ren C.D., Nofrey B., Teitelbaum S., Van Epps D.E. (2015). Histological Characterization of Human Breast Implant Capsules. Aesthetic Plast. Surg..

[43] Moore J.R. (1979). Applanation Tonometry of Breasts. Plast. Reconstr. Surg..

[44] Gylbert L.O. (1989). Applanation Tonometry for the Evaluation of Breast Compressibility. Scand. J. Plast. Reconstr. Surg. Hand Surg..

[45] Alfano C., Mazzocchi M., Scuderi N. (2004). Mammary Compliance: An Objective Measurement of Capsular Contracture. Aesthetic Plast. Surg..

[46] Brown T., Brown S., Murphy T. (2017). Breast Durometer (Mammometer): A Novel Device for Measuring Soft-Tissue Firmness and Its Application in Cosmetic Breast Surgery. Aesthetic Plast. Surg..

[47] Zahavi A., Sklair M.L., Ad-El D.D. (2006). Capsular Contracture of the Breast: Working towards a Better Classification Using Clinical and Radiologic Assessment. Ann. Plast. Surg..

[48] Wang D., Ai T., Xiong S., Zeng N., Wu M., Ren Y., Wu Y. (2021). 3D Images of the Silicone Implants in Capsular Contracture after Breast Augmentation Using Magnetic Resonance Imaging with SPACE Sequence. J. Plast. Reconstr. Aesthet. Surg..

[49] Georgieva M., Kammerer S., Prantl L., Jung F., Stroszczynski C., Jung E.M. (2020). Imaging of Breast Implant and Implant-Associated Complications: Capsular Contracture and Intra- or Extracapsular Rupture. Clin. Hemorheol. Microcirc..

[50] Larsen A., Timmermann A.M., Kring M., Mathisen S.B., Bak E.E.F., Weltz T.K., Ørholt M., Vester-Glowinski P., Elberg J.J., Trillingsgaard J. (2024). Development and Validation of a Diagnostic Histopathological Scoring System for Capsular Contracture Based on 720 Breast Implant Capsules. Aesthetic Surg. J..

[51] Lijmer J.G., Mol B.W., Heisterkamp S., Bonsel G.J., Prins M.H., van der Meulen J.H., Bossuyt P.M. (1999). Empirical Evidence of Design-Related Bias in Studies of Diagnostic Tests. JAMA.

[52] Sforza M., Kul Z., Saghir R., Saghir N., Okhiria R., Okhiria T., Sidhu M. (2023). Predicting the Expansion of the Lower Pole of the Breast Following Smooth Breast Implant Augmentation: A Novel Shear Wave Elastography Study. J. Plast. Reconstr. Aesthet. Surg..

[53] Fritsch H., Celik M., Warm M., Thangarajah F., Vogel-Minea C., Malter W., Pisek A., Eichler C. (2024). Sonographic Assessment of Breast Implants Using Strain Elastography and Shear Wave Elastography in an Animal Model. Anticancer. Res..

[54] Şanlı D.E.T., Şanlı A.N., Yıldırım D. (2026). Association Between Mastalgia and Elastographic Breast Stiffness in Patients with Subpectoral Silicone Implants: A Multiparametric Shear Wave Elastography Study. Acibadem Univ. Saglik. Bilim. Derg..

